# Elevated expression of UBE2T exhibits oncogenic properties in human prostate cancer

**DOI:** 10.18632/oncotarget.4712

**Published:** 2015-07-30

**Authors:** Mingxin Wen, Yongwon Kwon, Yongsheng Wang, Jian-Hua Mao, Guangwei Wei

**Affiliations:** ^1^ Department of Human Anatomy and Key Laboratory of Experimental Teratology, Ministry of Education, Shandong University School of Medicine, Jinan, Shandong, 250012 P.R. China; ^2^ Life Sciences Division, Lawrence Berkeley National Laboratory, Berkeley, CA 94127, USA

**Keywords:** UBE2T, prostate cancer, metastasis, vimentin

## Abstract

Increased expression of ubiquitin-conjugating enzyme E2T (UBE2T) is reported in human prostate cancer. However, whether UBE2T plays any functional role in prostate cancer development remains unknown. We here report the first functional characterization of UBE2T in prostate carcinogenesis. Prostate cancer tissue array analysis confirmed upregulation of UBE2T in prostate cancer, especially these with distant metastasis. Moreover, higher level of UBE2T expression is associated with poorer prognosis of prostate cancer patients. Ectopic expression of UBE2T significantly promotes prostate cancer cell proliferation, motility and invasion, while UBE2T depletion by shRNA significantly inhibits these abilities of prostate cancer cells. Xenograft mouse model studies showed that overexpression of UBE2T promotes whereas UBE2T depletion inhibits tumor formation and metastasis significantly. Collectively, we identify critical roles of UBE2T in prostate cancer development and progression. These findings may serve as a framework for future investigations designed to more comprehensive determination of UBE2T as a potential therapeutic target.

## INTRODUCTION

Prostate cancer (PCa) is the most frequent malignancy in men worldwide. The clinical behaviors range from slowly growing indolent tumors to highly aggressive, metastatic cancers. The pathologic stages of PCa begin with abnormal epithelial proliferation and prostatic intraepithelial neoplasia with progression to invasive carcinoma and eventually metastatic diseases [1]. While early localized disease is usually curable, the survival rate drops to only 34% with progression to invasive and metastatic disease [2]. For the initiation of PCa, researchers have found a number of genes or their alternations that promoted the progression of PCa [3–6]. However, with highly heterogeneous nature of PCa, unraveling the molecular and biological processes that contribute to PCa development and progression still remains as a challenging task.

We attempted to identify the aberrant expression of some genes in PCa using bioinformatics analysis of public available dataset and found that a novel gene *UBE2T* (ubiquitin-conjugating enzyme E2T) is significantly correlated with the disease-free survival of PCa patients. UBE2T is reported to participate in the DNA repair pathway and activate mono-ubiquitination of FANCD2 which is essential for the activation of FA core pathway [7, 8]. UBE2T has been also found overexpressed in lung [9, 10], bladder [11] and prostate cancers [12] and may act as oncogene-like gene in breast cancer by repressing BRCA1 expression and promoting the proliferation and transformation of breast cancer cells [13]. However, whether UBE2T plays any functional role in PCa has not been reported.

In this study, we focused on elucidating the significant role of UBE2T in prostate carcinogenesis. We showed that UBE2T is frequently found overexpressed in primary PCa, especially in patients with distant metastasis. Moreover, UBE2T expression level is correlated with poor patient disease-free survival. We further demonstrated that UBE2T promotes proliferation, invasion, tumor formation and metastasis of PCa cells. These data indicated that UBE2T is a novel oncogene and a potential therapeutic target for PCa.

## RESULTS

### Overexpression of UBE2T in prostate cancers is positively correlated with metastasis and poor prognosis

**Figure 1:**
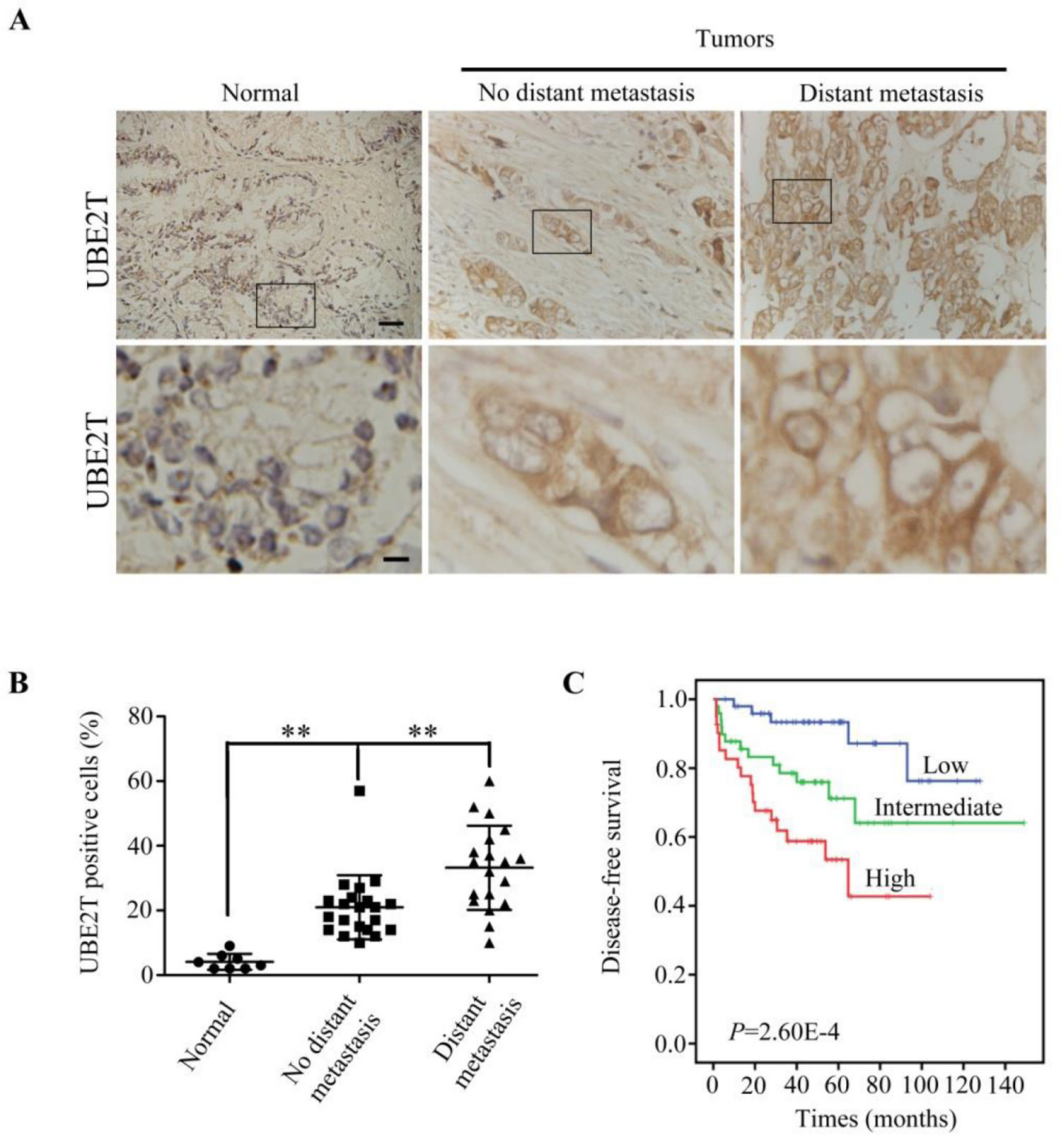
UBE2T is overexpressed and related to the prognosis of prostate cancer patients **A.** Immunohistochemical analysis of UBE2T levels in different stages of prostate cancer in a tissue array. From left to right are representative images of UBE2T expression in normal tissue, prostate cancer tissues without or with metastasis. Normal: normal tissues; No distant metastasis: cancer tissues without distant metastasis; Distant metastasis: cancer tissues with distant metastasis. Lower panels are the higher magnification of indicated areas in upper panel. **B.** Analysis of UBE2T positive cells in the above prostate cancer tissue array. **C.** Survival analysis of patients with prostate cancers in a publically available dataset (GSE21032). Scale bars, 100 μm (A upper panel) and 20 μm (A lower panel). **P < 0.01 in panel B based on the Student *t* test. Error bars, SD.

We first confirmed whether UBE2T expression level was elevated in PCa by immunochemical staining of UBE2T in a tissue microarray. As shown in Figure 1A, UBE2T expression was hardly detectable in normal prostate tissue, but overexpressed in PCa tissue and most highly expressed in metastatic PCa tissue. Quantification analyses showed significant differences among different status of PCa (Figure 1B).

To evaluate whether UBE2T was related with prognosis of PCa patients, we carried out the bioinformatics analysis of the publicly available dataset (GSE21032). The PCa patients were divided into tertiles based on UBE2T expression levels (low = bottom tertile, intermediate = middle tertile, and high = top tertile). It was found that the patients with higher UBE2T mRNA level in PCa tissues had poorer disease free survival (DFS) than those with lower UBE2T expression level (Figure 1C) suggesting that UBE2T expression significantly correlated with the prognosis of PCa patients.

### UBE2T promotes prostate cancer cell proliferation

**Figure 2:**
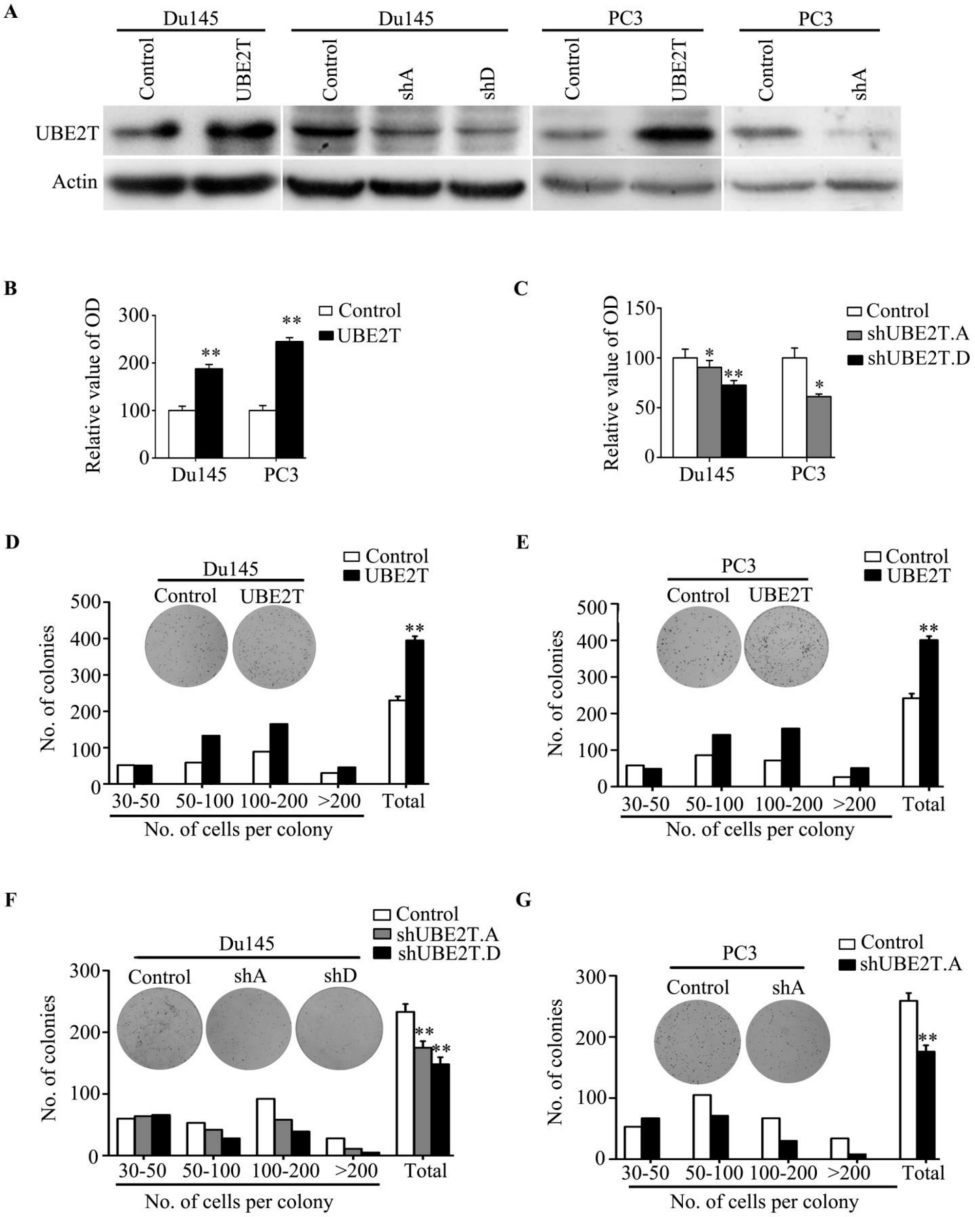
UBE2T promotes proliferation of prostate cancer cells **A.** Western Blot analysis of UBE2T levels in the established cell lines. **B.** MTT assay shows the obviously increased proliferative rate in PC3 and Du145 expressing pBabe-UBE2T. **C.** MTT assay shows the decrease of proliferative rate in cells expressing pSuper-shUBE2T. **D.** and **E.** Colony formation assay demonstrates an increase in the number of clones by overexpression of UBE2T in PC3 and Du145. **F.** and **G.** Colony formation assay demonstrates a decrease in number of clones by knockdown of UBE2T. shA and shD for shUBE2T.A and D respectively. *P < 0.05, **P < 0.01 based on the Student *t* test. Error bars, SD.

To better understand the role of UBE2T in PCa, we used retroviral vectors to establish PCa cell lines stably overexpressing or silencing UBE2T. The expression levels of UBE2T in the subsequent cell lines were examined by Western blotting (Figure 2A and Supplementary Figure S1A). We first used 3-(4,5-dimethylthiazol-2-yl)-2, 5-diphenyltetrazolium bromide (MTT) and colony formation assays to investigate a growth-promoting effect of UBE2T on PCa cells. MTT assay revealed that Du145, PC3 and LNCaP cells with overexpression of UBE2T proliferated more rapidly than their corresponding control cells (Figure 2B and Supplementary Figure S1B). In colony formation assay, overexpression of UBE2T in Du145 (Figure 2D), PC3 (Figure 2E) and LNCaP (Supplementary Figure S1C) cells significantly increased the numbers and sizes of clones. In contrast, silencing UBE2T expression by two shRNAs targeting UBE2T (shUBE2T A and D) in Du145 and PC3 cells dramatically suppressed the growth (Figure 2C, 2F and 2G) of both cell lines in a dose-dependent manner as shUBE2T.D suppressed the growth more dramatically than shUBE2T.A which are in concordance with the knockdown efficacy on UBE2T in Du145 cells.

### UBE2T enhances the tumor growth in prostate cancer xenograft mouse model

**Figure 3:**
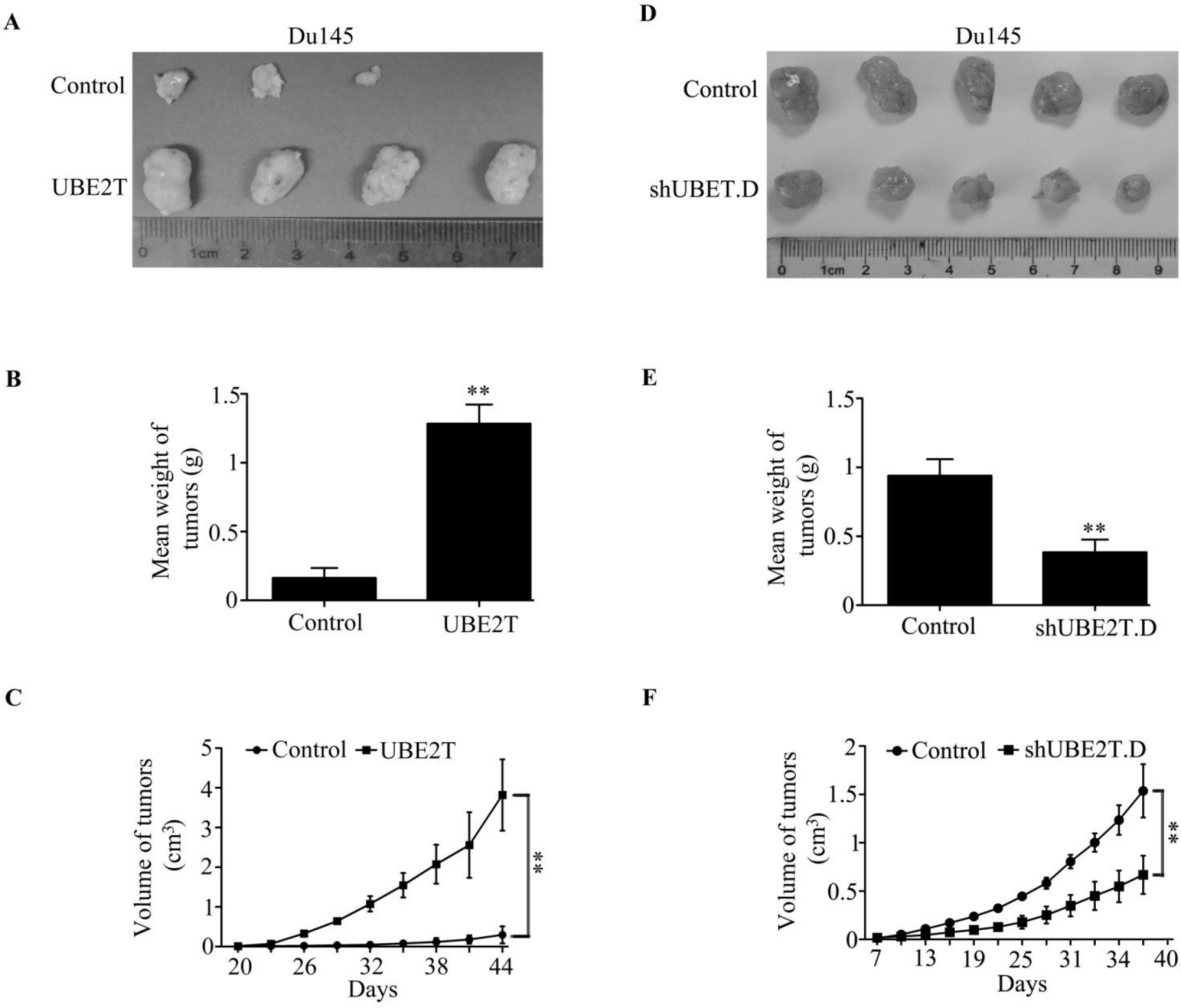
UBE2T promotes tumor growth of prostate cancer cells *in vivo* One million of Du145 cells with ectopic expression of UBE2T **A–C.** or five million cells with UBE2T silence **D–E.** were injected into nude mice subcutaneously. 60 days after inoculation each tumor was taken for picture (A and D) and weight measurement (B and D). Tumor growth from each cell lines was measured each 3 days since the appearance of tumors and tumor volume was calculated and plotted **C.** and **F.** *n* = 5 for each cell lines. **P < 0.01 based on the Student *t* test. Error bars, SD.

In order to confirm whether the growth-promoting effect of UBE2T observed in cultured cells is relevant to prostate tumor growth *in vivo*, Du145 cells with ectopic or silent expression of UBE2T were subcutaneously inoculated into BALB/C athymic mice respectively. Overexpression of UBE2T significantly accelerated tumor growth (Figure 3A) and induced an increase in tumor weight (Figure 3B) and volume (Figure 3C). In contrast, silencing UBE2T expression inhibited tumor growth (Figure 3D) and induced a decrease in tumor weight (Figure 3E) and volume (Figure 3F). Taking all together, we concluded that UBE2T promotes the proliferation of PCa cells *in vivo* and *in vitro*.

### UBE2T induces epithelial-mesenchymal transition in prostate cancer cells

**Figure 4:**
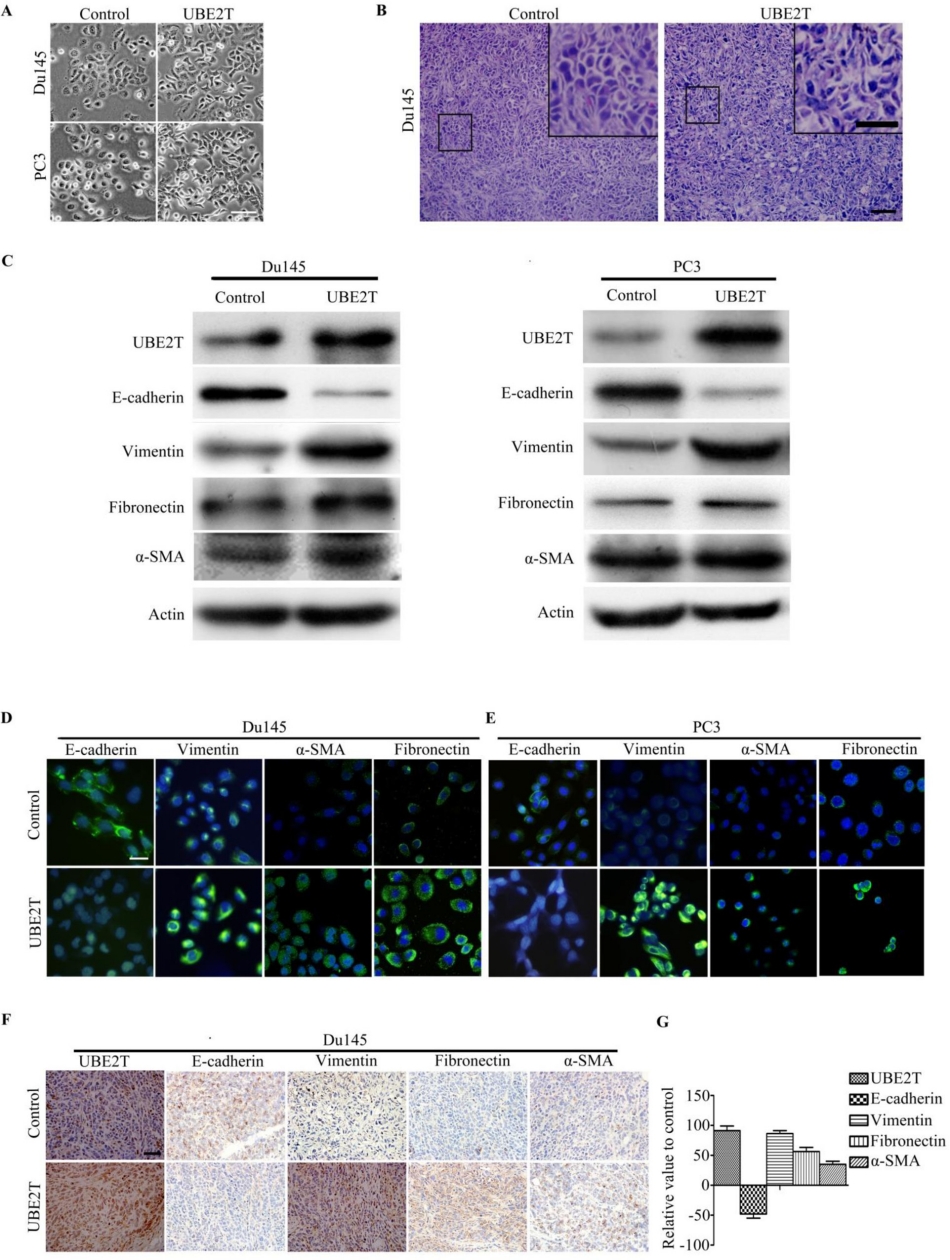
UBE2T induces EMT of prostate cancer cells **A.** Micrographs showing the morphology of Du145, PC3 with overexpression of UBE2T and their corresponding control cells. **B.** H&E staining of xenograft tumor sections. **C.** Western blot analysis of the expression of the epithelial cell marker E-cadherin and the mesenchymal cell markers vimentin, fibronectin and alpha-smooth muscle actin in PC3 and Du145 cells overexpressing UBE2T. **D.** and **E.** Immunofluorescence images of EMT markers in Du145 and PC3 overexpressing UBE2T. **F.** Images of immunochemistry staining for UBE2T and EMT markers in sections of xenograft tumors from Du145 cells with or without overexpression of UBE2T. **G.** Positive cells in (F) were counted and plotted in histogram. Scale bars: 50 μm (A), 100 μm (B and F) and 50 μm (inserts in B), 20 μm (D and E).

We observed changes in cell morphology after UBE2T overexpression. Under microscope, both Du145 and PC3 cells overexpressing UBE2T showed mesenchymal morphology compared with control cells (Figure 4A). By H&E staining for tumors formed in xenograft mouse model, we found that tumor cells overexpressing UBE2T showed mesenchymal-like morphology (Figure 4B). This indicated that UBE2T possibly induces epithelial-mesenchymal transition (EMT) of PCa cells. To further confirm these observations, we assessed protein markers of EMT. As shown in Figure 4C, downregulation of epithelial cell marker (E-cadherin) and upregulation of mesenchymal cell markers (vimentin, fibronection and alpha-smooth muscle action) were detected by Western blotting in UBE2T overexpressing cells, and the same results were found through immunofluorescence analyses (Figure 4D and 4E). We further confirmed this EMT marker expression pattern change in xenograft tumor sections through immunochemical staining (Figure 4F and 4G). Moreover, we found similar expression pattern of EMT marker in androgen-dependent LNCaP cells with overexpression of UBE2T by Western blotting (Supplementary Figure S2A). These results collectively indicated that UBE2T promotes EMT of PCa cells.

### UBE2T accelerates prostate cancer cell migration and invasion

**Figure 5:**
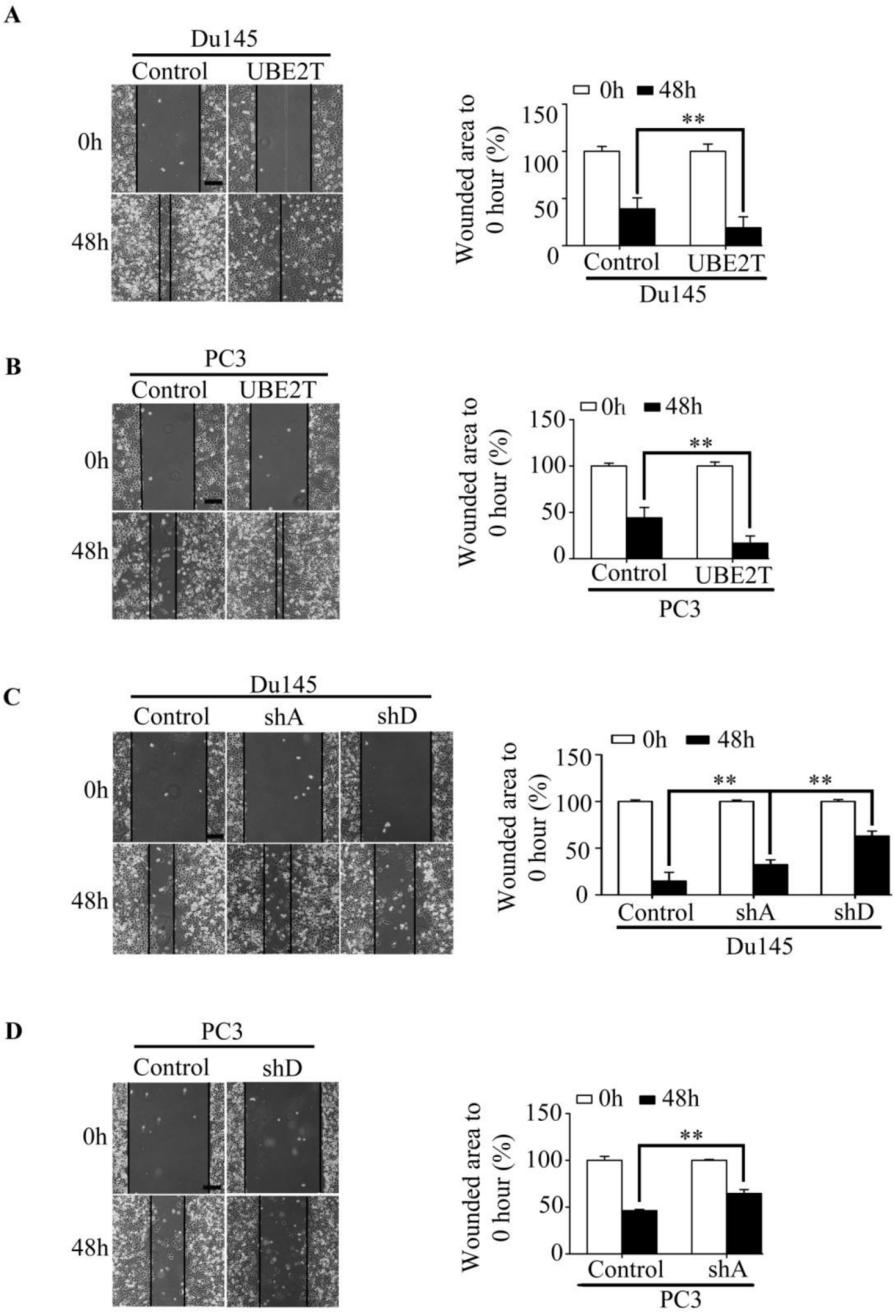
UBE2T promotes migration of prostate cancer cells **A–B.** Wound healing assay revealed a faster wound-healing speed in PC3 and Du145 expressing UBE2T and the unhealed area was measured and the values were shown in histogram. The initiative unhealed area was used as a 100% control. **C–D.** Wound healing assay revealed a depressed speed in both cell lines with silent UBE2T expression, and the unhealed area was measured and the values were shown in histogram. The initiative unhealed area was used as a 100% control. shA and shD for shUBE2T. A and D respectively. Scale bars: 100 μm. **P < 0.01 based on the Student *t* test. Error bars, SD.

**Figure 6:**
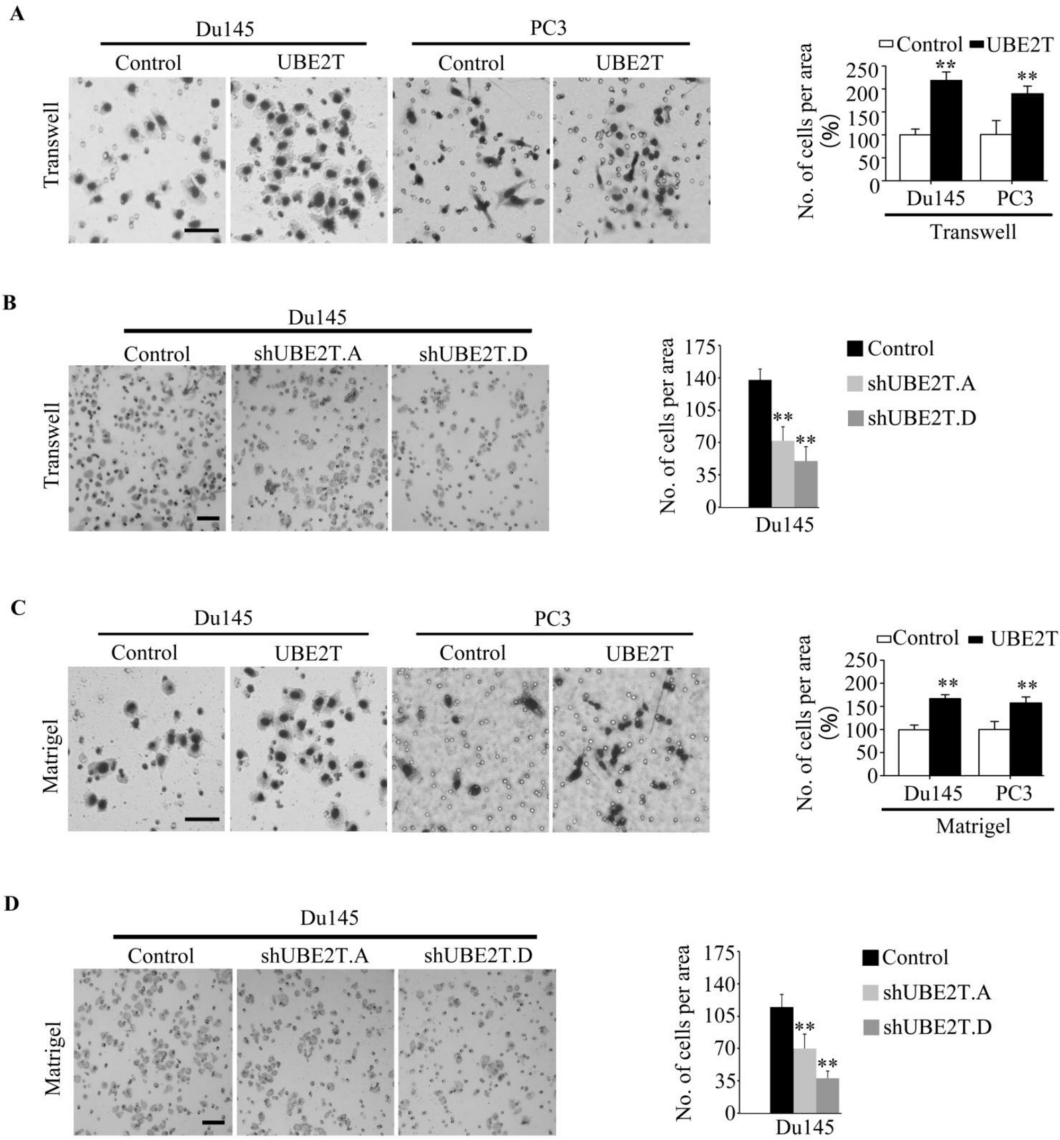
UBE2T promotes the mobility of prostate cancer cells **A.** PC3 and Du145 expressing UBE2T possessed more migrating abilities in traswell assay. The migrated cells were plotted as the average number of cells in five random fields. **B.** Du145 with silent expression of UBE2T possessed less migrating abilities in traswell assay. The migrated cells were plotted as the average number of cells in five random fields. **C.** PC3 and Du145 expressing UBE2T possessed more invaded abilities in matrigel assay. The invaded cells were plotted as the average number of cells in five random fields. **D.** Du145 with silent expression of UBE2T possessed less invaded abilities in matrigel assay. The invaded cells were plotted as the average number of cells in five random fields. Scale bars: 100 μm (A and C), 50 μm (B and D). **P < 0.01 based on the Student *t* test. Error bars, SD.

We then evaluated the effects of UBE2T on migration and invasion of PCa cells. Scratch assay revealed that UBE2T significantly accelerated wound healing of Du145 (Figure 5A), PC3 (Figure 5B) and LNCaP (Supplementary Figure S2B) cells while silencing UBE2T decreased the rate of migration (Figure 5C and 5D). To further confirm the role of UBE2T in migration, transwell assay was carried out. Overexpression of UBE2T promoted more cells migrated through the membrane to the bottom of the aperture (Figure 6A and Supplementary Figure S2C). In contrast, silencing UBE2T expression restrained this progress (Figure 6B). Moreover, matrigel assay was used to evaluate the invasive potential of PCa cells with altered UBE2T expression. As shown in Figure 6C and Supplementary Figure S2C, ectopic expression of UBE2T significantly enhanced the invaded rate of PCa cells while silencing UBE2T expression decreased the number of invaded PCa cells (Figure 6D). These results revealed that UBE2T promotes migration and invasion of PCa cells.

### UBE2T promotes prostate cancer cell metastasis *in vivo*

**Figure 7:**
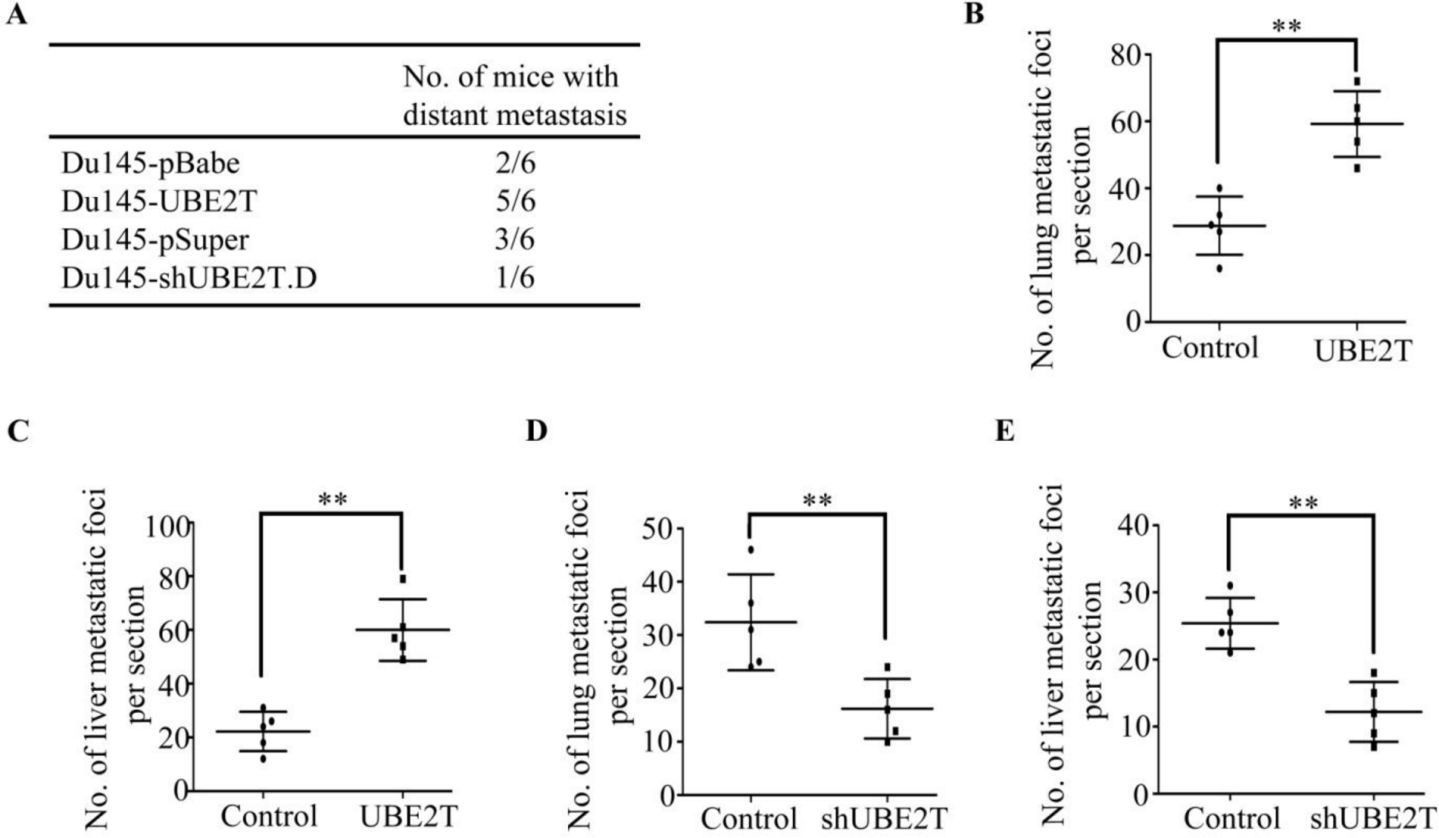
UBE2T promotes metastasis of prostate cancer *in vivo* **A.** The total numbers of mice with distant metastasis at 60 days after injection of Du145 cells with ectopic or silent expression of UBE2T into tail vein. **B.** and **C.** The numbers of metastatic foci per section in lung (B) and liver (C) of individual mouse with injection of Du145 cells with overexpression of UBE2T. **D.** and **E.** the numbers of metastatic foci per section in lung (D) and liver (E) of individual mouse with injection of Du145 cells with silent expression of UBE2T. **P < 0.01 based on the Student *t* test. Error bars, SD.

To examine whether the function of UBE2T in migration and invasion *in vitro* was relevant to metastasis of PCa cells *in vivo*, Du145 cells with ectopic or silent UBE2T expression were inoculated into tail vein of BALB/C athymic mice respectively. 60 days later, we observed that more mice injected with PCa cells overexpressing UBE2T had distant metastasis (Figure 7A). In addition, more metastasis foci in lung (Figure 7B) and liver (Figure 7C) were counted in each mouse injected with PCa cells overexpressing UBE2T. On the contrary, silencing UBE2T expression decreased the number of metastatic mice (Figure 7A) and metastatic foci in lung (Figure 7D) and liver (Figure 7E).

Collectively, both *in vivo* and *in vitro* data strongly showed the biological role of UBE2T as a promoter of tumor growth and an inducer of EMT and metastasis in PCa.

### A positive correlation between UBE2T and vimentin expression in human prostate cancer

**Figure 8:**
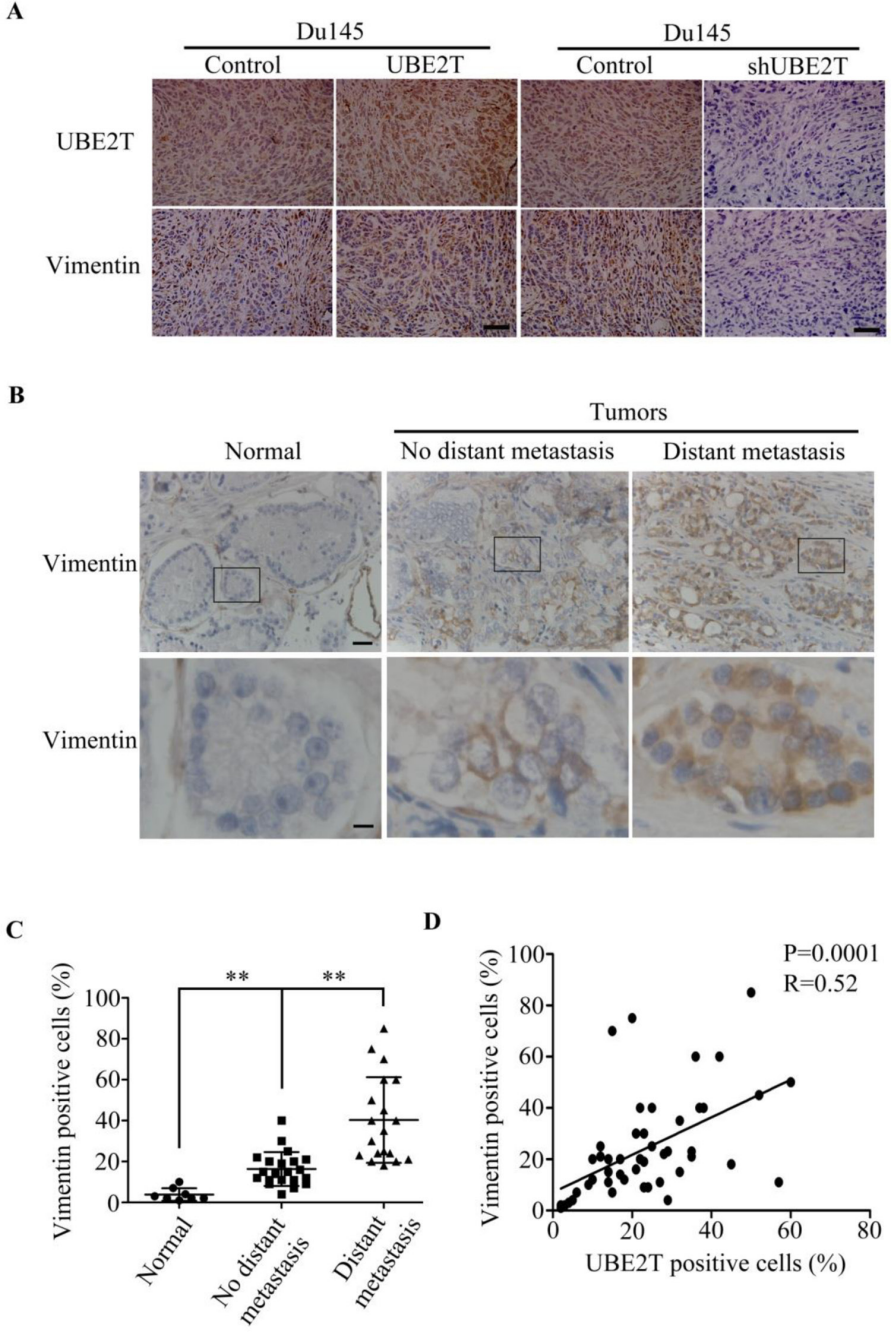
UBE2T expression level is positively associated with vimentin expression **A.** Immunohistochemical analysis of UBE2T and vimentin expression in xenograft prostate tumors. **B.** Immunohistochemical analysis of vimentin level in a human prostate cancer tissue array as shown in Figure 1. From left to right are representative images of vimentin expression in normal tissue, cancer tissues without and with distant metastasis. Lower panels are the higher magnification of indicated areas in upper panel. **C.** Analysis of vimentin positive cells in normal tissue, cancer tissues without and with distant metastasis in tissue array. **D.** Correlation analysis between UBE2T and vimentin levels Lower panels are the higher magnification of indicated areas in upper panel in this tissue array. Normal, normal tissues; No distant metastasis, cancer tissues without distant metastasis; Distant metastasis, cancer tissues with distant metastasis. Scale bars, 100 μm (A upper panel and B) and 20 μm (B, lower panel). **P < 0.01 based on the Student *t* test. Error bars, SD.

As shown above, we found that vimentin expression level is elevated in the PCa cells with overexpressing UBE2T (Figure 4C and Supplementary Figure S2A). The same observation was also found in xenograft prostate tumors (Figure 8A left panel). In contrast, silencing UBE2T expression decreased vimentin expression level in PCa cells (Figure 8A right panel). To further confirm the relationship between UBE2T and vimentin, vimentin expression was analyzed in a tissue array. As shown in Figure 8B, vimentin expression was found high in PCa tissues with distant metastasis, moderate in PCa tissues without distant metastasis and low in normal prostate tissue, indicating vimentin expression was associated with metastasis of PCa (Figure 8C). In linear correlation analysis, UBE2T expression level was positively correlated with that of vimentin (Figure 8D).

## DISCUSSSION

UBE2T is a new member of ubiquitin-conjugating enzymes [14]. This study, for first time, indicated that UBE2T acts as an oncogene in PCa. We found that UBE2T expression is elevated in PCa and higher level of UBE2T is associated with poorer prognosis of PCa patients. Overexpression of UBE2T in PCa cells robustly promotes cell proliferation, migration and invasion *in vitro* and tumor growth and metastasis *in vivo*. Of note, we discovered a positive correlation between UBE2T and vimentin expression, both expression levels of UBE2T and vimentin are associated with the malignant properties of prostate tumors.

UBE2T was previously reported as essential for the monoubiquitination of FANCD2 protein which was the key step of DNA repair in Fancini anemia [7]. The putative role of UBE2T as an oncogene in cancer development was supported by the observations that aberrant expression of UBE2T in bladder, lung and prostate cancers [10–11]. The fact that UBE2T locates at 1q32.1 and the gain of 1q can been observed in most cancers [15–18], which may result in the increased expression of UBE2T, leads further support to the possibility of UBE2T as an oncogene. Perhaps the most convincing evidence is that downregulation of BRCA1 by UBE2T in breast cancer significantly promoted proliferation and tumor growth of breast cancer cells. Consistent with these reports, we showed that UBE2T overexpression promotes PCa cell proliferation and enhanced tumor growth *in vivo*. More important, we pointed out a novel functional role of UBE2T in metastasis by regulating EMT of PCa cells.

In this study, PCa cells with overexpression of UBE2T displayed a mesenchymal phenotype with the enhanced potentials of migration and invasion *in vitro*. Consistent with the notion that EMT is essential for tumor cells separating from the solid tumors and invading into distant sites, all of these characteristics induced by UBE2T *in vitro* culminated to increased numbers of distant metastases *in vivo*. These experimental findings are consistent with the clinical observation that high UBE2T expression level is correlated with aggressive clinical stage and shorter disease free survival.

Multiple EMT regulators have been extensively reported. Among them, vimentin is the mammalian intermediate filament proteins that is ubiquitously expressed in normal mesenchymal cells [19] and often used as a marker of cells undergoing EMT during both normal development and metastatic progression. In this study, we revealed that both UBE2T and vimentin expressions were closely related with metastatic abilities of PCa and there is a positive relationship between UBE2T and vimentin through linear correlation analysis. Interestingly, UBE2T elevated vimentin expression in PCa cells. Since vimentin was proved to be a regulator of cell adhesion by affecting formation and turnover adhesion structures [20–25] and associated with cancer migration [26, 27], we speculate vimentin may be a potential downstream protein of UBE2T in UBE2T-induced EMT and metastasis. Considering that UBE2T is an ubiquitin-conjugating enzyme, we speculate that UBE2T may upregulate vimentin level through a more complex indirect mechanism. Further determination of the interaction between UBE2T and vimentin may not only provide more details to the function of UBE2T, but may also reveal a novel therapeutic target in PCa by disrupting the interaction between UBE2T and vimentin.

UBE2T may be a novel indicator of PCa along with about 900 000 new PCa patients diagnosed every year [28]. Nowadays, the most important diagnostic and prognostic indicators of PCa are stage, pre-therapy PSA level, and Gleason score (GS) [29]. Unfortunately, misdiagnosis was unavoidable on account of that PCA3 is not only specially overexpressed in PCa [30, 31] but also elevated in the presence of prostate disorders [30] and over-diagnosis was made because of the limitation of GS which is given to PCa based upon its microscopic appearance [28]. Either misdiagnosis or over-diagnosis could cause people experience the side effects of treatment. Interestingly, UBE2T is only overexpressed in PCa, and plays an important role in PCa metastasis which is the main cause of PCa death, UBE2T could be a better diagnostic and prognostic indicator of PCa patients.

In summary, we have for the first time achieved that UBE2T overexpression was sufficient to induce EMT of PCa cells as well as promote tumor growth and metastasis *in vivo*. We also preliminarily revealed that UBE2T acts as an oncogene, at least in part, through cooperating with vimentin. Together with all the results, our studies suggested UBE2T as a promising prognostic and therapeutic target in PCa.

## MATERIALS AND METHODS

### Cell culture and retroviral transduction

PCa cell lines PC3, Du145 and LNCaP were maintained in RPMI-1640 (HyClone) with L-glutamine. Phoenix packaging cell and 293T were maintained in high glucose DMEM medium (HyClone). All medium was supplemented with 10 percents fetal bovine serum (TBD) and 1 percent penicillin/streptomycin (Solarbio). The cell lines were incubated at 37°C in 5% CO_2_.

Human gene UBE2T cDNA was cloned into retroviral vector pBabe-puro and shRNA (A and D) specific for UBE2T was cloned into retroviral vector pSuper-puro. Supernatants containing pBabe, pSuper, pBabe-UBE2T, pSuper-sh.UBE2T.A and pSuper-sh.UBE2T.D were produced in Phoenix packaging cells. We respectively transfected PC3 and Du145 with these different viral supernatants containing 4 μg/ml polybrene (Sigma). Then cells were selected with puromycin (2 μg/ml) and cell lines containing PC3-pBabe, PC3-pSuper, Du145-pBabe, Du145-pSuper, PC3-pBabe-UBE2T, PC3-pSuper-sh.UBE2T.A, Du145-pBabe-UBE2T, Du145-pSuper-sh.UBE2T.A, and Du145-pSuper-sh.UBE2T.D were retrovirously established.

### RNA isolation and reverse transcription PCR

Cells were incubated in 60 mm dishes (Corning) and Trizol (Invitrogen) was added at 70% density after PBS washing followed RNA isolation according to trizol introductions. After concentration detection of total RNA extraction, 3 μg RNA was pipetted for RT-PCR (Applied Biosystems, Thermal Cycler) of UBE2T using the primers: forward primer 5′-CAAATATTAGGTGGAGCCAACAC-3′ and reverse primer 5′-TAGATCACCTTGGCAAAGAACC-3′, and agarose gel electrophoresis followed at 120 V for 30 min.

### Proliferation assay

MTT (3-[4, 5-dimethylthiazol-2-yl]-2, 5-diphenyl-tetrazolium bromide, Solarbio, M8180) assay and Colony Formation were used to detect the proliferative rate of UBE2T-overexpression or UBE2T-knockdown cell lines. For MTT assay, 1000 cells were plated into 96-well plate and cultured at 37°C in a. MTT was dropped at 70% density and the 96-well plate was incubated for 4 hours followed 150 μl DMSO dropped after removing the supernatant carefully. The OD values were measured by the machine (Multiskan 3). In Colony formation, 800 cells were plated into 60 mm dishes and incubated for 2 weeks in a homogeneous atmosphere with 5% CO_2_ at 37°C. Then the clones were fixed by methal alcohol and) stained for 30 minutes in Giemsa (Sigma, GS-500) followed ddH_2_O washed. The clones were counted under a microscope.

### Western-blot assay

Western-blot assay was used to analyze the expressions of UBE2T and EMT (epithelial-mesenchymal transition) markers of indicated cell lines. Cells were cultured in 100 mm dishes (Corning) and RIPA buffer (Beyotime) containing protein inhibitor cocktail was input at 70–90% density. Whole protein was extracted by centrifugation (14000 rpm) for 20 minutes at 4°C. Samples containing 30–50 ug of protein were separated by SDS-polyacrylamide gel electrophoresis. Proteins were transferred to PVDF (polyvinylidene fluoride) membrane (Millipore) which was blocked in 5% BSA (Bovine Serum Albumin) or 5% skim milk. Then the membrane was probed overnight at 4°C in blocking buffer with primary antibodies (Actin 1:2000, E-cadherin 1:1000, Vimentin 1:1000, Fibronectin 1:5000, UBE2T 1:1000) followed by washing in TBST (0.02M Tris PH 7.6, 0.8% NaCl, 01% Tween-20) and incubated in TBST containing secondary antibodies (1:10000) for 1 hour at RT. After washing in TBST again, the chemiluminescence liquid (Millipore) was added and the fluorescence was captured by photographic film (Kodak) or FluorChem E (Proteinsimple, FE0444).

### Migration assay

Scratch assay and transwell assay to detect the migrated ability. Scratch assay was performed by a 200 μl pipette in 60 mm dishes when cells grew up to 90–100% density. And the floated cells were removed with twice Hank's (Beyotime, C0218) washes, and wound pictures were taken at 0 hour, 24 hours and 48 hours by Olympus IX70 inverted microscope. In transwell assay, bottom chamber was filled with 1 ml RPMI-1640 with 10% fetal bovine serum and 1% penicillin/streptomycin and the top chamber was filled with the same media with 4 × 10^4^ (PC3 cells with ectopic expression of UBE2T) or 2 × 10^4^ (Du145 cells with ectopic expression of UBE2T) or 4 × 10^4^ (LNcaP cells with ectopic expression of UBE2T) cells suspended. The cells were incubated for 48 hours and the fixed in methanol longer than 30 minutes and stained with Giemsa stain after the cells on the top surface of membrane removed by cotton swab. The pictures were taken by Olympus IX70 inverted microscope and the numbers of migrated cells were counted.

### Invasion assay

The chambers (BD, 3097) were coated with matrigel (BD, 354605) and incubated for longer than 30 minutes at 37°C to perform invasion assay. The bottom chambers were filled with 1 ml RPMI-1640 with 10% fetal bovine serum and 1% penicillin/streptomycin and the top chamber were filled with 200 μl RPMI-1640 without FBS or antibiotics but with 10^5^ (PC3 cells with ectopic expression of UBE2T) or 6 × 10^4^ Du145 cells with ectopic expression of UBE2T or 8 × 10^4^ (LNcaP cells with ectopic expression of UBE2T) suspended cells. Cells were incubated in the incubator for 48 hours. The non-invaded cells on the top surface of membrane were removed by cotton swab and the invaded cells were fixed in methanol longer than 30 minutes and stained with Giemsa stain for 30 minutes. The pictures were taken by Olympus IX70 inverted microscope. The invaded cells were counted.

### Immunofluorescence

Cells with altered expression of UBE2T were cultured on glass coverslips (NEST, 801007) in 24-well plate and fixed by 4% paraformaldehyde for 15 min at 50–60% density followed by washing in PBS. After blocking in goat serum (1:10 in PBS) for 30 min, the coverslips were incubated with primary antibodies (diluted in primary stain diluting buffer, Beyotime, P0103) overnight at 4°C and secondary antibodies 1 hour at 37°C. Nuclei were visualized by 4,6-diamidine-2-phenylindole staining (DAPI, Solarbio, D8200). The coverslips touched face down a drop of Anti-fade Mounting Medium (Beyotime, P0126) on a slide and the fluorescence was captured by laser scanning confocal microscopy.

### Immunohistochemical staining

Immunohistochemical staining was performed on formalin-fixed, paraffin-embedded tissues or prostate array from Alenabio, China PR483a. Endogenous peroxidase activity was blocked with 3% hydrogen peroxide. Antigen retrieval was carried out in citrate buffer (10 mM, pH-6) for 20 min at more than 92°C. Tissue sections were incubated first with the primary antibodies of UBE2T (1:50, Cell Signaling Technology), vimentin (1:50, Cell Signaling Technology) for 16 h at 4°C and then incubated for 30 minutes at 37°C and then subsequently with a secondary biotinylated antibody (SP-9000, China) for 30 minutes at 37°C followed by incubation with streptavidin–peroxidase complex for 5 min at room temperature.

### *In vivo* tumor growth and metastasis

Nude mice were purchased from Shanghai Slac Laboratory Animal Co. Ltd. and maintained in microisolator cages. All animals were used in accordance with institutional guidelines and the current experiments were approved by the Use Committee for Animal Care. For subcutaneous inoculation, different numbers of tumor cells (1 million Du145-UBE2T and control cells, 5 million Du145-shUBE2T and control cells) resuspended in PBS medium and inoculated subcutaneously into 8-weekold nude mice. The tumors were measured every 3 days after appearance of tumors and the tumor volume was calculated by the formula length × width2/2. The mice were killed 44 days after the inoculation. For metastasis assays, cells were resuspended in PBS at a concentration of 3 × 10^7^ cells/ml. Cell suspension (0.1 ml) was injected into tail veins of nude mice. All of the mice were killed by CO_2_ 60 days after inoculation.

### Statistical analysis

Statistical analysis Data were described as the mean ± SD. Association between UBE2T and vimentin expression in prostate tissue microarray was assessed using the Spearman rank correlation test. DFS was estimated using the Kaplan–Meier method. The relationship between survival period and each of the variables was analyzed using the log-rank test for categorical variables. Comparisons between different groups were undertaken using the Student two-tailed *t* test. The criterion of statistical significance was *P* < 0.05. Statistical analysis was done with SPSS/Win11.0 software (SPSS Inc.).

## SUPPLEMENTARY FIGURES


