# Supplementary material for: Elevated expression of UBE2T exhibits oncogenic properties in human prostate cancer
Source: Oncotarget. 2015 Jul 30;6(28):25226–39. doi: 10.18632/oncotarget.4712 (PMC4694827; doi:10.18632/oncotarget.4712)
Supplement: Supplementary file 1 [file oncotarget-06-25226-s001.pdf]

SUPPLEMENTARY FIGURES

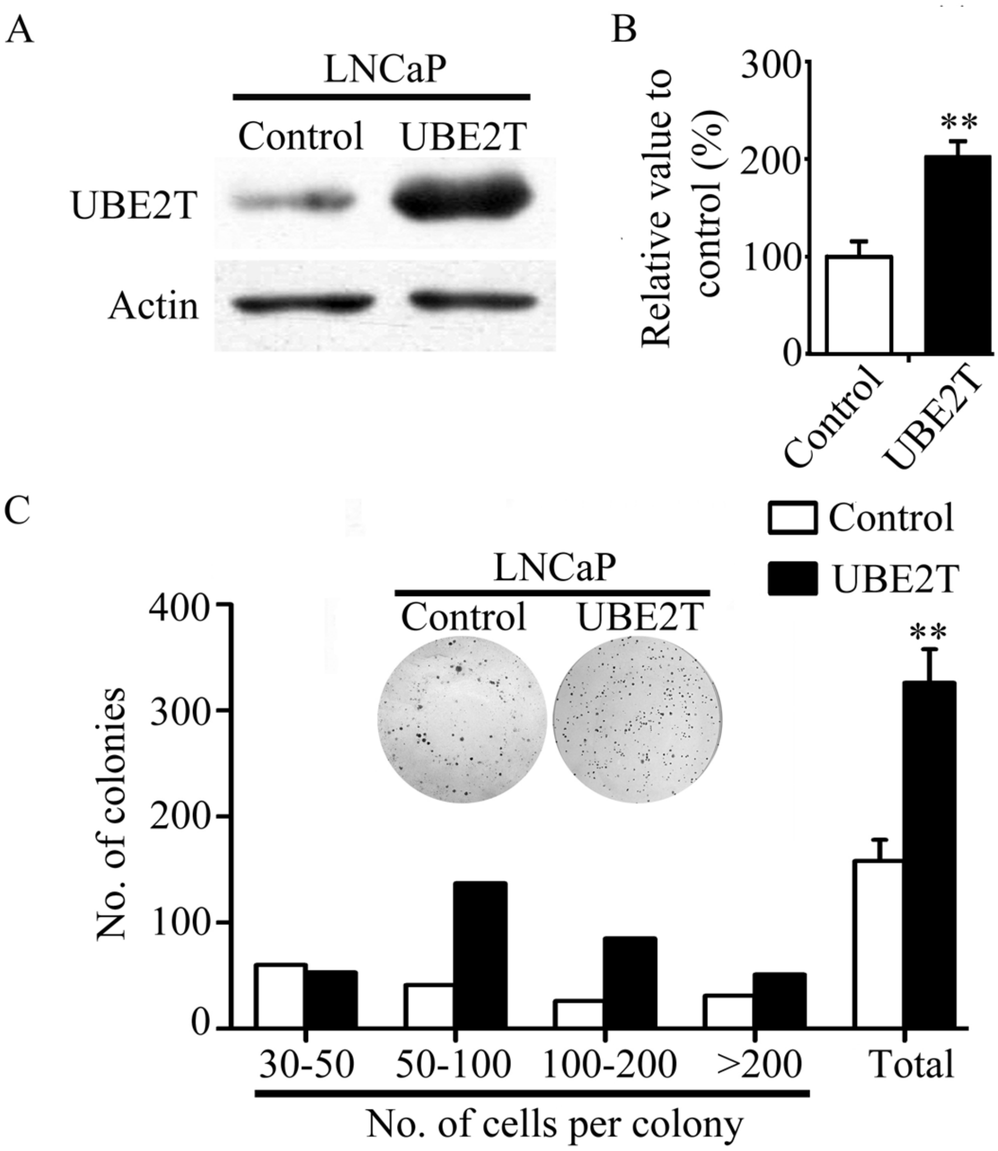

Supplementary Figure S1: UBE2T promotes proliferation of LNCaP cells. A. Western Blot analysis of UBE2T levels in LNCaP cells overexpressing UBE2T. **B.** MTT assay showed the obviously increased proliferative rate of LNCaP cells with expression of UBE2T. **C.** Colony formation assay demonstrated an increase in the number of clones by overexpression of UBE2T in LNCaP cells. ***P* < 0.01 based on the Student *t* test. Error bars, SD.

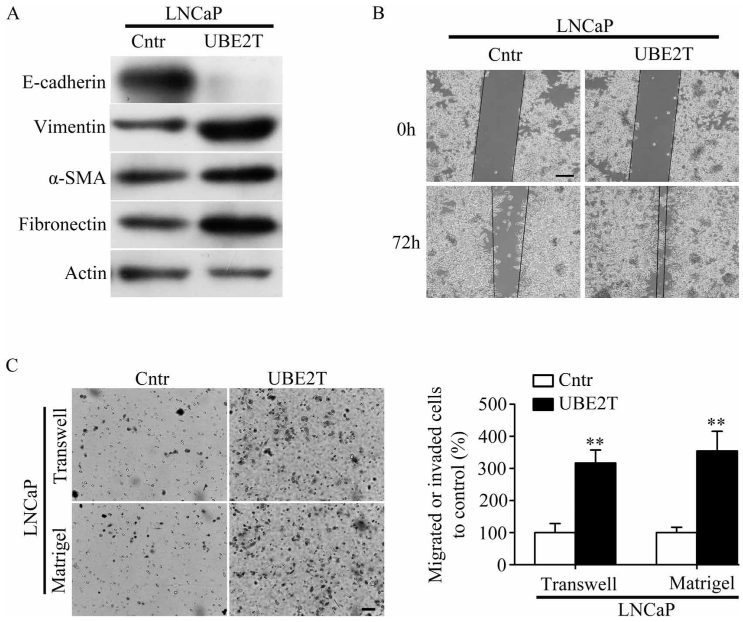

Supplementary Figure S2: UBE2T promotes migration and invasion of LNCaP cells. A. Western blot analysis of EMT markers in LNCaP cells with overexpression of UBE2T. **B.** Wound healing assay revealed a faster wound healing rate in LNCaP overexpressing UBE2T. **C.** LNCaP cells overexpressing UBE2T possessed more migrating ability in transwell assay and more invading ability in matrigel assay. Scale bars: 100 μm (B) and 50 μm (C) $^{**}P < 0.01$ based on the Student *t* test. Error bars, SD.
